# Psychological and Psychiatric Events Following Immunization with Five Different Vaccines against SARS-CoV-2

**DOI:** 10.3390/vaccines10081297

**Published:** 2022-08-11

**Authors:** Mario García-Alanis, Marisa Morales-Cárdenas, Liz Nicole Toapanta-Yanchapaxi, Erwin Chiquete, Isaac Núñez, Santa Elizabeth Ceballos-Liceaga, Guillermo Carbajal-Sandoval, Carla Toledo-Salinas, David Alejandro Mendoza-Hernández, Selma Cecilia Scheffler-Mendoza, José Antonio Ortega-Martell, Daniel Armando Carrillo-García, Noé Hernández-Valdivia, Alonso Gutiérrez-Romero, Javier Andrés Galnares-Olalde, Fernando Daniel Flores-Silva, José Luis Díaz-Ortega, Gustavo Reyes-Terán, Hugo López-Gatell, Ricardo Cortes-Alcalá, José Rogelio Pérez-Padilla, Antonio Arauz, Miguel García-Grimshaw, Sergio Iván Valdés-Ferrer

**Affiliations:** 1 Department of Neurology and Psychiatry, Instituto Nacional de Ciencias Médicas y Nutrición Salvador Zubirán, Mexico City 14080, Mexico; 2 Department of Internal Medicine, Instituto Nacional de Ciencias Médicas y Nutrición Salvador Zubirán, Mexico City 14080, Mexico; 3 Dirección General de Epidemiología, Secretaría de Salud, Mexico City 01480, Mexico; 4 Comisión Coordinadora de Institutos Nacionales de Salud y Hospitales de Alta Especialidad, Mexico City 14610, Mexico; 5 Instituto Nacional de Pediatría, Mexico City 04530, Mexico; 6 Universidad Autónoma del Estado de Hidalgo, Pachuca 42039, Mexico; 7 Department of Neurology, Instituto Nacional de Neurología y Neurocirugía Manuel Velasco Suárez, Mexico City 14269, Mexico; 8 Centro Nacional para la Salud de la Infancia y la Adolescencia, Secretaría de Salud, Mexico City 14080, Mexico; 9 Secretaría de Salud, Gobierno de México, Mexico City 01480, Mexico; 10 Instituto Nacional de Enfermedades Respiratorias Ismael Cosío Villegas, Mexico City 14080, Mexico; 11 Hospital General Tijuana, Tijuana 22000, Mexico; 12 Department of Infectious Diseases, Instituto Nacional de Ciencias Médicas y Nutrición Salvador Zubirán, Mexico City 14080, Mexico; 13 Feinstein Institutes for Medical Research, Manhasset, NY 11030, USA

**Keywords:** immunization stress-related responses, anxiety, panic attack, fear, vaccines, SARS-CoV-2

## Abstract

Background: Despite the high number of vaccines administered against severe acute respiratory syndrome coronavirus 2 (SARS-CoV-2) worldwide, the information on the psychological/psychiatric adverse events following immunization (AEFI) with these newly developed vaccines remains scarce. Objective: To describe the frequency of psychological/psychiatric symptoms among recipients of five different anti-SARS-CoV-2 vaccines and to explore the factors associated with their development reported in the nationwide Mexican registry of AEFI against SARS-CoV-2. Methods: Descriptive study of all the psychological/psychiatric symptoms, including anxiety, panic attacks, insomnia, and agitation reported to the Mexican Epidemiological Surveillance System from 21 December 2020 to 27 April 2021, among adult (≥18 years old) recipients of 7,812,845 doses of BNT162b2, ChAdOx1 nCov-19, rAd26-rAd5, Ad5-nCoV, or CoronaVac. The factors associated with their development are determined by multivariate regression analysis. Results: There were 19,163 AEFI reports during the study period; amongst them, 191 (1%) patients had psychological/psychiatric symptoms (median age of 41 years, interquartile range of 32–54; 149 [78%] women) for an observed incidence of 2.44 cases per 100,000 administered doses (95% confidence interval [CI] 2.12–2.82), 72.8% of psychiatric AEFIs were reported among recipients of BNT162b2. The median time from vaccination to symptom onset was 35 min (interquartile range: 10–720). Overall, the most common psychological/psychiatric symptoms were anxiety in 129 (67.5%) patients, panic attacks in 30 (15.7%), insomnia in 25 (13%), and agitation in 11 (5.7%). After adjusting for the confounding factors, the odds for developing psychological/psychiatric symptoms were higher for those concurrently reporting syncope (odds ratio [OR]: 4.73, 95% CI: 1.68–13.33); palpitations (OR: 2.47, 95% CI: 1.65–3.70), and dizziness (OR: 1.59, 95% CI: 1.10–2.28). Conclusion: In our population, psychological/psychiatric symptoms were extremely infrequent AEFIs. No severe psychiatric AEFIs were reported. Immunization stress-related responses might explain most of the detected cases.

## 1. Introduction

Since the first case of severe acute respiratory syndrome coronavirus 2 (SARS-CoV-2) infection, the etiological agent of coronavirus 2019 disease (COVID-19) was reported on 31 December 2019, where at least 430 million cases have been reported globally, resulting in no less than 5.9 million COVID-19-related deaths worldwide [1]. At present, large-scale anti-SARS-CoV-2 immunization campaigns remain the most effective intervention method to overcome the ongoing pandemic [2].

Due to the number of anti-SARS-CoV-2 vaccines in use, Mexico is in a unique position to evaluate the differences between multiple vaccines based on three different platforms: mRNA (BNT162b2), adenovirus vectored (ChAdOx1 nCov-19, rAd26-rAd5, and Ad5-nCoV), and inactivated whole-virion vectored (CoronaVac) [3]. Despite the growing evidence of vaccine safety and effectiveness, limited information exists about the psychological/psychiatric adverse events following immunization (AEFI) against SARS-CoV-2. Despite the effectiveness of the five vaccines included in this report, many systemic AEFIs or vaccination-related issues have not been fully described [4,5,6], including, psychological/psychiatric issues.

Some psychological/psychiatric AEFIs might present as immunization stress-related responses (ISRRs), which is an entity that includes a broad range of signs and symptoms that may arise around immunization-related anxiety and not the vaccine product, a defect in the quality of the vaccine, or an error of the immunization program [7,8]. Usually, ISRRs occur when new vaccines are introduced or when changes to an established immunization program are performed, and these can be disruptive to immunization programs by reducing public trust and impacting vaccination coverage [9]. In addition, the accelerated development process and authorization of COVID-19 vaccines have raised concerns regarding their safety and encouraged vaccine hesitancy. At the time of writing this manuscript, psychological/psychiatric symptoms following immunization against SARS-CoV-2 were not systematically evaluated in large cohorts. Hence, from a nationwide registry, we aim to describe the frequency of psychological/psychiatric symptoms and explore the factors associated with their development among recipients of different vaccines against SARS-CoV-2 who reported an AEFI during the first five months of the Mexican anti-COVID-19 immunization program.

## 2. Material and Methods

### 2.1. Study Design and Population

A descriptive study evaluating the psychologic and psychiatric AEFIs reported following the first and second doses of the BNT162b2, ChAdOx1 nCov-19, rAd26-rAd5, Ad5-nCoV, and CoronaVac anti-SARS-CoV-2 vaccines from 24 December 2020 to 27 April 2021, in Mexico. For this, we analyzed a dataset of AEFIs obtained from the General Board of Epidemiology of the Mexican Ministry of Health. Mexico started the anti-SARS-CoV-2 vaccination program on 24 December 2020. From that day, data on all AEFIs were captured through a passive epidemiological surveillance system where events were reported either by the attending physician or directly by the vaccine recipient. After the reports were received, all AEFIs were characterized as either serious or non-serious according to the World Health Organization operational definition at the local level. The operational details of the Mexican epidemiological system and case definitions have been published elsewhere [10,11,12,13].

For the present analysis, we included adult (≥18 years old) vaccine recipients—at the cutoff time, the Mexican Immunization Program had not yet started with those younger than 18 years old—who reported an AEFI following the first or second dose of the different anti-SAR-CoV-2 vaccines. Cases of serious neurologic AEFIs (e.g., acute stroke, Guillain–Barré syndrome, acute transverse myelitis, and peripheral facial palsy) or any other primarily neurologic manifestations were excluded.

### 2.2. Standard Protocol Approvals, Registrations, and Patient Consent

The study protocol was approved by the Ethics and Research Committees of Instituto Nacional de Ciencias Médicas y Nutrición Salvador Zubirán and the Mexican Ministry of Health (Ref. NER-3903-21-22-1). Due to the study’s observational nature and the use of a de-identified dataset, both committees waived the need for signed informed consent.

### 2.3. Data Collection and Definitions

We obtained a de-identified dataset of all AEFIs, including age, sex, occupation, history of recent non-SARS-CoV-2 infection (≤15 days before immunization), allergies, current or past SARS-CoV-2 infections confirmed either by real-time reverse-transcription-polymerase chain reaction (RT-PCR) or antigen testing, as well as vaccine-associated adverse events as assessed by the attending physician. Pre-existing medical and psychiatric comorbidities were not routinely recorded, limiting their use. The dataset also included the interval from vaccine administration to first-symptom onset and clinical outcome.

The frequency and type of symptoms were recorded from a direct non-structured interview collected on the vaccination site by medical personnel at the healthcare posts in each vaccination unit. Clinical notes were electronically recorded at each medical encounter. We extracted the frequency and types of psychological/psychiatric symptoms from these electronic clinical notes included in the de-identified dataset, including anxiety, panic attacks, transient paresthesia, fear, insomnia, agitation, and/or sadness. Two board-certified psychiatrists (M.G.-A. and M.M.-C.) reviewed and verified all the data. Keywords were used as search terms to find possible psychological/psychiatric symptoms within the database to analyze each case among all the possible identified symptoms. The search terms used were anxiety, anxiety crisis, nervousness, depression, fear, panic attack, sadness, psychomotor agitation, psychosis, hallucination/s, delusion, mania, hypomania, irritability, joy, and happiness.

### 2.4. Statistical Analysis

Categorical variables were reported as frequencies and proportions; continuous variables were reported as median with interquartile range (IQR) or as mean with standard deviation (SD). Categorical variables were analyzed with Pearson *X*^2^ or Fisher’s exact test. After testing normality with the Kolmogorov–Smirnov test, the continuous variables were analyzed with the Mann–Whitney U test or Student’s *t*-test. Observed incidences for each vaccine subtype per 1,000,000 administered doses with 95% confidence intervals (CIs) were calculated using the Wilson method [14]. We performed a multivariate analysis by binary logistic regression analysis to determine the factors associated with the development of psychological/psychiatric symptoms adjusted for age, sex, clinical, type of vaccine, and signs and symptoms of reactogenicity. The results are reported as odds ratios (ORs) with a 95% CI. The model’s fitness was evaluated using the Hosmer–Lemeshow goodness-of-fit test and considered as reliable when the resulting *p*-value was >0.20. All *p*-values were two-tailed and considered significant with a *p*-value < 0.05. All analyses were performed with IBM SPSS Statistics, version 26 (IBM Corp., Armonk, NY, USA).

## 3. Results

During the study period, the Mexican Epidemiological Surveillance System received and processed 19,163 AEFI reports among recipients of 7,812,845 doses of any approved vaccine for an observed AEFI incidence of 245.3 cases per 100,000 doses (95% CI: 241.8–248.8). Of all reports, we excluded 127 (0.66%) serious AEFIs (neurologic and non-neurologic). Among the remaining 19,036 cases, we detected 191 patients with reports of psychological/psychiatric symptoms, representing 1% of all AEFIs or 2.44 (95% CI: 2.12–2.82) cases per 100,000 administered doses.

Among those with psychological/psychiatric symptoms, the median age was 41 years (IQR: 32–54) years (Table 1); 149 (78%) were women and 110 (57.6%) were healthcare workers (HCWs). The median time from vaccination to first AEFI symptom was 35 min (IQR: 10–720 min) in the psychological/psychiatric symptoms group, shorter than in those with other events. The proportion of patients with a history of SARS-CoV-2 infection, recent non-SARS-CoV-2 infection, or allergies was similar between groups. Other systemic symptoms referred by the patients with psychological/psychiatric symptoms were headache (54.9%), dizziness (43.5%), generalized weakness or fatigue (38.7%), palpitations/racing heart (29.8%), and myalgias (26.1%). The frequencies of dizziness (43.5% vs. 27.9%, *p* = 0.001); palpitations/racing heart (29.8% vs. 11.3%, *p* < 0.001); syncope (2.6 % vs. 0.6%, *p* < 0.001); and nausea (34% vs. 24.7%, *p* = 0.004) were significantly higher among the recipients who presented psychological/psychiatric symptoms than those who did not (Table 2).

**Figure 1:**
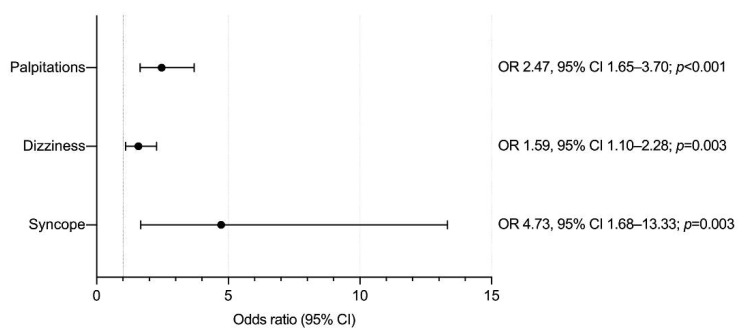
Factors associated with developing psychological/psychiatric symptoms as an AEFI among recipients of five different vaccines against SARS-CoV-2. Abbreviations: OR, odds ratio, CI, confidence interval. Model adjusted for age, sex, medical history, timing from vaccination, and reported symptoms.

The most common psychological/psychiatric symptoms were anxiety in 129 (67.5%) patients; panic attacks in 30 (15.7%); insomnia in 25 (13%); and agitation in 11 (5.7%). Their main characteristics are described in Table 3. HCWs reported psychological/psychiatric symptoms (*n* = 110; 57.6% vs. *n* = 81; 42.4%). Other lower-frequency symptoms were fear (*n* = 2) and sadness (*n* = 3). After adjusting for confounding factors by multivariable analysis, the odds for reporting psychological/psychiatric symptoms were significantly higher for those reporting syncope (OR: 4.73, 95% CI: 1.68–13.33); palpitations/racing heart (OR: 2.47, 95% CI: 1.65–3.70); and dizziness (OR: 1.59, 95% CI: 1.10–2.28) (Figure 1). None of the other included variables were associated with developing psychological/psychiatric symptoms.

## 4. Discussion

Our data indicate that SARS-CoV-2 vaccine recipients may experience psychological/psychiatric symptoms following vaccination, including anxiety, panic attacks, insomnia, agitation, depression, and fear. In general, psychological/psychiatric AEFIs were observed in recipients to all vaccines; however, in our dataset, 72.8% of these AEFIs occurred in recipients of BNT162b2, a group comprising only 51.4% of injected doses during the study period. However, psychological/psychiatric AEFIs are extremely unusual across all five vaccines, and this apparent imbalance towards BNT162b2 may be a reflection of receiving a vaccine with a vector (mRNA) with—by then—limited clinical experience, probably generating more anxiety among its early recipients.

There are few reports of psychiatric/psychological symptoms associated with these vaccines. A cross-sectional study evaluating 1415 United States-based HCWs who responded to a survey after being immunized with mRNA-vectored vaccines (either BNT162b2 or mRNA-1273), reported decreased sleep quality, anxiety, mood changes, depression, and behavioral changes [4]. In a prospective survey study on AEFIs following the first dose of either BNT162b2 or ChAdOx1 nCov-19 vaccines among South Korean HCWs, anxiety was far more common and, interestingly, more frequent among recipients of ChAdOx1 nCov-19 than BNT162b2 (22.4% vs. 4.7%, respectively; *p* < 0.001) [15].

In our study, the relative frequencies of the different psychiatric/psychological symptoms were lower than in the previous studies. We hypothesized that two factors could explain the differences observed between the studies. On the one hand, we relied on a passive report system where symptoms may not have been reported unless considered meaningful by the patient or the caregiver, whereas, in active surveillance systems, patients will prompt symptoms regardless of their perceived severity. On the other hand, medical and psychological/psychiatric comorbidities were not routinely recorded on the dataset and were only identified in those who looked for medical care due to a putative AEFIs. Hence, our result is more likely to reflect the symptoms and signs that are medically meaningful to the vaccine recipients to prompt them to seek medical advice. Finally, the cultural and individual differences beyond methodological discrepancies (e.g., perception of vaccine benefit versus harm) may also add to the observed divergence of symptoms.

Interestingly, as can be observed by multivariate analysis, there was no association between having a history of allergy or acute reactogenicity (e.g., headache, dizziness, asthenia, palpitations, and myalgia) in patients reporting psychological/psychiatric AEFIs than among those reporting other types of (but not including psychological/psychiatric) AEFIs. Additionally, we detected that anxiety, panic attacks, and agitation commonly occurred within the first 20 min after vaccination, probably related to an increased awareness of real or perceived adverse reactions. Among all psychological/psychiatric symptoms, anxiety, panic attacks, insomnia, and agitation were more frequent in females than in males. Possible explanations for these findings include increased reactogenicity in female recipients, higher awareness and reporting rates among females, and sexual dimorphism of the immune response [16].

The sexual dimorphism of the immune response has been implicated as a mechanism in other types of AEFIs, usually attributed to estrogen-dependent hormonal processes, which may be crucial for the immune response [17]. There is an association with a higher immediate antigen response in the innate immune system of females, as an increased level of type I interferons (IFN-I) innate immune response, T-cell response, and epigenetic changes [18]. Women present more robust immune responses to some vaccines, which has been observed in hepatitis B and influenza vaccines [19]. A study that evaluated over 25 million vaccine administrations reported that anaphylaxis was slightly more common in women; this predominance was not statistically significant [20]. COVID-19 vaccines have become a game changer, especially when implicating mRNA platforms, with some surveillance data on BNT162b2 and mRNA-1273 vaccine-related anaphylaxis reporting a solid female predominance, with up to 95% of the cases [21], implying that there are other underlying mechanisms, such as previous exposure to the excipients of the vaccines, which may contribute to the effect of sexual dimorphism [17].

Different mechanisms can explain the presence of psychological/psychiatric symptoms. One of them is the widespread anxiety levels associated with COVID-19 and its vaccines, and the perception of an increased risk of adverse reactions and its related negative consequences (e.g., anxiety, fear, difficulties in concentration, insomnia, fatigue, and constantly checking the news and social media), or a history of sickness behavior associated with reactogenicity [22]. Moreover, individual variability can also play an important role as some individuals developing psychological/psychiatric may immediately seek medical attention, while others may wait or may not seek help at all [23]. This represents the result of different interacting variables, including those related to COVID-19 vaccines (including perceived efficacy and safety), individual characteristics (psychological stress, mental health, personality, coping mechanisms, and socioeconomics), social (access to and trust in verifiable sources versus unverified and potentially fake sources of information), and healthcare systems (providing information and reassurance) [24].

Despite the efficacy and safety of several COVID-19 vaccines, public concerns about the potential adverse reactions associated with vaccines are still widespread [25,26,27,28], particularly the fear of developing AEFI [29]. Stress related to SARS-CoV-2 vaccines decreases over time as safety data have emerged. A recent Chinese study reported that the risk factors for stress responses included younger age, lower education level, history of chronic diseases, mistrust in vaccine efficacy, having experienced vaccine-related allergic events, having had COVID-19, and history of mental illness symptoms [30].

The underlying factors associated with vaccine hesitancy are multiple and context-specific, varying across time and socio-demographic variables [31]. COVID-19 vaccine-related psychological stress has been associated with hesitancy or refusal to be vaccinated [28]. Reducing psychological stress associated with COVID-19 vaccination would hypothetically foster a confidence in and acceptance of vaccination in vulnerable populations [32].

ISRRs are recognized by the Council of International Medical Sciences (CIOMS) and the WHO working group in pharmacovigilance as one of the five categories of AEFI [33], including a range of symptoms and signs that may arise after immunization, such as stress-related psychiatric reactions or disorders. These events are unrelated to the vaccine product, a defective vaccine, or an immunization program-related error. Two types of mass anxiety-related AEFIs have been described: anxiety reactions present shortly after the vaccination, and symptoms including dizziness, fainting, headache, hyperventilation, and weakness. A motor reaction typically has a slower onset after the vaccination and presents with motor agitation, twitching, motor tics, speech impairment, gait disturbances, and sometimes functional seizures [9].

For some COVID-19 vaccine recipients, the combination of pain and stressful situations may manifest in a range of symptoms and signs consistent with a stress response. Needle-injection phobia is one of the most prevalent mental disorders and can result in the avoidance of preventive measures and treatment, as observed during influenza vaccination campaigns [34]. A stress response involves a combination of biological factors occurring within an individual with his or her psychological strengths, vulnerabilities, and knowledge/preparedness, all occurring within a particular social context [7]. In some countries, these conditions have led to significant public concerns and reduced vaccine coverages [35].

Our study had several limitations. First, this was a cross-sectional study; hence, we were unable to establish a causal effect. Second, we used a dataset in which no psychiatric or medical background could be retrieved. Therefore, we were unable to detect patients with depression, anxiety, or other psychological/psychiatric symptoms before vaccination. Third, the dataset did not specify first or second dosing; hence, we were unable to attribute a higher risk to either dosing schedule. In addition, the reporting of AEFIs depended entirely on the patient’s reference and the physician’s accuracy in recognizing and accurately reporting psychological/psychiatric AEFIs. Most of the symptoms reported above only occurred in the early post-vaccination phase; our database failed to recognize long-term (>30 days) vaccine-related AEFIs. Additionally, although we identified some risk factors, a proper psychiatric follow-up was not available. The strengths of our study included its large sample size and the inclusion of recipients of five different vaccines, including vaccines for which psychological/psychiatric safety data were either incomplete or have not been previously reported.

We cannot overemphasize how critical it is to differentiate between acute psychological/psychiatric and anaphylactic AEFIs. Misrecognizing one from the other may lead to potentially life-threatening clinical interventions. While the treatment for anaphylaxis requires urgent life-preserving interventions, such as the prompt administration of intramuscular epinephrine, ISRRs can spontaneously resolve and do not put the patient in immediate danger.

In conclusion, our data indicate that all vaccines are associated with non-serious, transient psychological/psychiatric AEFIs that are extremely infrequent and likely to be multifactorial. We hope that this report helps to reduce stress responses, psychological/psychiatric symptoms, and hesitancy associated with COVID-19 vaccines.

## Figures and Tables

**Table 1:** Baseline characteristics according to the development of psychiatric events.

	Psychiatric AEFI (*n* = 191)	Non-Psychiatric AEFI (*n* = 18,972)	Total (*n* = 19,163)
Age, median (IQR), years	41 (32–54)	40 (30–52)	40 (17–101)
Sex, *n* (%)			
Male	42 (22)	4673 (24.3)	4715 (24.6)
Female	149 (78)	14,299 (74.6)	14,448 (75.3)
Occupation, *n* (%)			
Healthcare worker	110 (57.6)	11,971 (62.46)	12,081 (63)
Non-healthcare worker	81 (42.4)	7001 (36.53)	7082 (37)
Medical history, *n* (%)			
History of SARS CoV-2 infection	37 (19.4)	4362 (23)	4399 (23)
Non-SARS CoV-2 infection (<15 days)	3 (1.6)	296 (1.6)	299 (1.6)
Allergy (any)	83 (43,4)	7772 (40.9)	12,038 (62.8)
Time to AEFI report, median (IQR), minutes	35 (10–720)	300 (15–1440)	300 (15–1440)
Vaccine type, *n* (%)			
BNT162b2	139 (72.8)	15,047 (78.5)	15,186 (79.2)
ChAdOx1 nCov-19	20 (10.5)	1800 (9.4)	1820 (9.5)
Ad5-nCoV	15 (7.9)	945 (4.9)	960 (5)
CoronaVac	11 (5.8)	972 (5.1)	983 (5.1)
rAd26-rAd5	6 (3.1)	414 (2.6)	420 (4.2)

Abbreviations: IQR, interquartile range; SARS-CoV-2, severe acute respiratory syndrome coronavirus.

**Table 2:** Adverse events following vaccination according to the development of psychiatric events.

	Psychiatric AEFI (*n* = 191)	Non-Psychiatric AEFI (*n* = 18,972)	Total (*n* = 19,163)
Adverse events following vaccination, *n* (%)			
Fever, 38 °C *n* (%)	30 (15.7)	5024 (26.2)	5054 (26.4)
Dizziness, *n* (%)	83 (43.5)	5352 (27.9)	5435 (28.4)
Palpitations/racing heart, *n* (%)	57 (29.8)	2175 (11.4)	2232 (11.6)
Syncope, *n* (%)	5 (2.6)	106 (0.6)	111 (0.6)
Nausea, *n* (%)	65 (34)	4739 (24.7)	4804 (25.1)
Myalgia, *n* (%)	50 (26.2)	6860 (35.8)	6910 (36.1)
Bronchospasm, *n* (%)	5 (2.6)	190 (1)	195 (1)
Headache, *n* (%)	105 (54.9)	11,623 (60.7)	11,728 (61.2)
Vomiting, *n* (%)	22 (11.5)	1531 (8)	1553 (8.1)
Arthralgia, *n* (%)	47 (24.6)	5646 (29.5)	5693 (29.7)
Rhinorrhea, *n* (%)	16 (8.4)	2142 (11.2)	2158 (11.3)
Chills, *n* (%)	39 (20.4)	4531 (23.6)	4570 (23.8)
Diarrhea, *n* (%)	15 (7.9)	1827 (9.5)	1842 (9.6)
Adynamia, *n* (%)	41 (21.5)	4418 (23.1)	4459 (23.3)
Asthenia, *n* (%)	74 (38.7)	7387 (38.5)	7461 (38.9)

**Table 3:** Baseline characteristics of the cases.

	Anxiety (*n* = 129)	Panic Attack (*n* = 30)	Insomnia (*n* = 25)	Agitation (*n* = 11)
Age, median (IQR), years	40 (31–53)	49 (37–64)	45 (32–64)	35 (29–57)
Sex, *n* (%)				
Male	25 (19.4)	6 (20)	12 (48)	1 (9.1)
Female	104 (80.6)	24 (80)	13 (52)	10 (90.9)
Occupation *n* (%)				
Physician	28 (21.7)	3 (10)	1 (4)	2 (18.2)
Nurse	48 (37.2)	2 (6.7)	7 (28)	7 (63.6)
Emergency medical technician	10 (7.8)	4 (13.3)	2 (8)	0 (0)
Non-healthcare related	43 (33.3)	21 (70)	15 (60)	2 (18.2)
Medical history, *n* (%)				
History of SARS CoV-2 infection	28 (21.7)	4 (13.3)	2 (6)	2 (18.2)
Non-SARS CoV-2 (≤15 days)	2 (1.6)	0 (0)	0 (0)	1 (9.1)
Allergies (any)	61 (47.3)	10 (33.3)	11 (44)	7 (63.6)
Time from vaccination to psychological symptoms onset, median (IQR), minutes	20 (10–690)	18 (10–60)	360 (120–1440)	40 (3–2280)
Timing from vaccination to psychological symptoms, *n* (%)				
0–5 min	23 (17.8)	6 (20)	0 (0)	3 (27.3)
6–10 min	18 (14.0)	6 (20)	0 (0)	2 (18.2)
11–20 min	24 (18.6)	7 (23.3)	0 (0)	0 (0)
21–40 min	4 (3.1)	2 (6.7)	0 (0)	1 (9.1)
41–60 min	4 (3.1)	2 (6.7)	0 (0)	0 (0)
1–24 h	42 (32.6)	5 (16.7)	20 (80)	1 (9.1)
>1–7 days	8 (6.2)	2 (6.7)	4 (16)	0 (0)
>7 days	1 (3.9)	0 (0)	1 (4)	4 (36.4)
Vaccine type, *n* (%)				
BNT162b2	100 (77.5)	16 (53.3)	14 (56)	10 (90.9)
ChAdOx1 nCov-19	10 (7.8)	6 (20)	7 (28)	0 (0)
CoronaVac	9 (7.0)	1 (3.3)	1 (4)	0 (0)
Ad5-nCoV	5 (3.9)	6 (20)	3 (12)	1 (9.1)
rAd26-rAd5	5 (3.9)	1 (3.3)	0 (0)	0 (0)
Number of dose, *n* (%)				
First	98 (76.6)	24 (80)	20 (80)	8 (72.7)
Second	31 (23.4)	6 (20)	5 (20)	3 (27.3)

Abbreviations: IQR, interquartile range, SARS-CoV-2, severe acute respiratory syndrome coronavirus 2.

## Data Availability

The manuscript provides all the collected data. De-identified data supporting the findings of this study are available to qualified researchers upon written request from the corresponding author.
